# Supplementary material for: Epithelial Cell Adhesion Molecule (EpCAM) Expression in Human Tumors: A Comparison with Pan-Cytokeratin and TROP2 in 14,832 Tumors
Source: Diagnostics (Basel). 2024 May 17;14(10):1044. doi: 10.3390/diagnostics14101044 (PMC11120328; doi:10.3390/diagnostics14101044)
Supplement: Supplementary file 1 [file diagnostics-14-01044-s001.zip › Supplementary Figure 4.pdf]

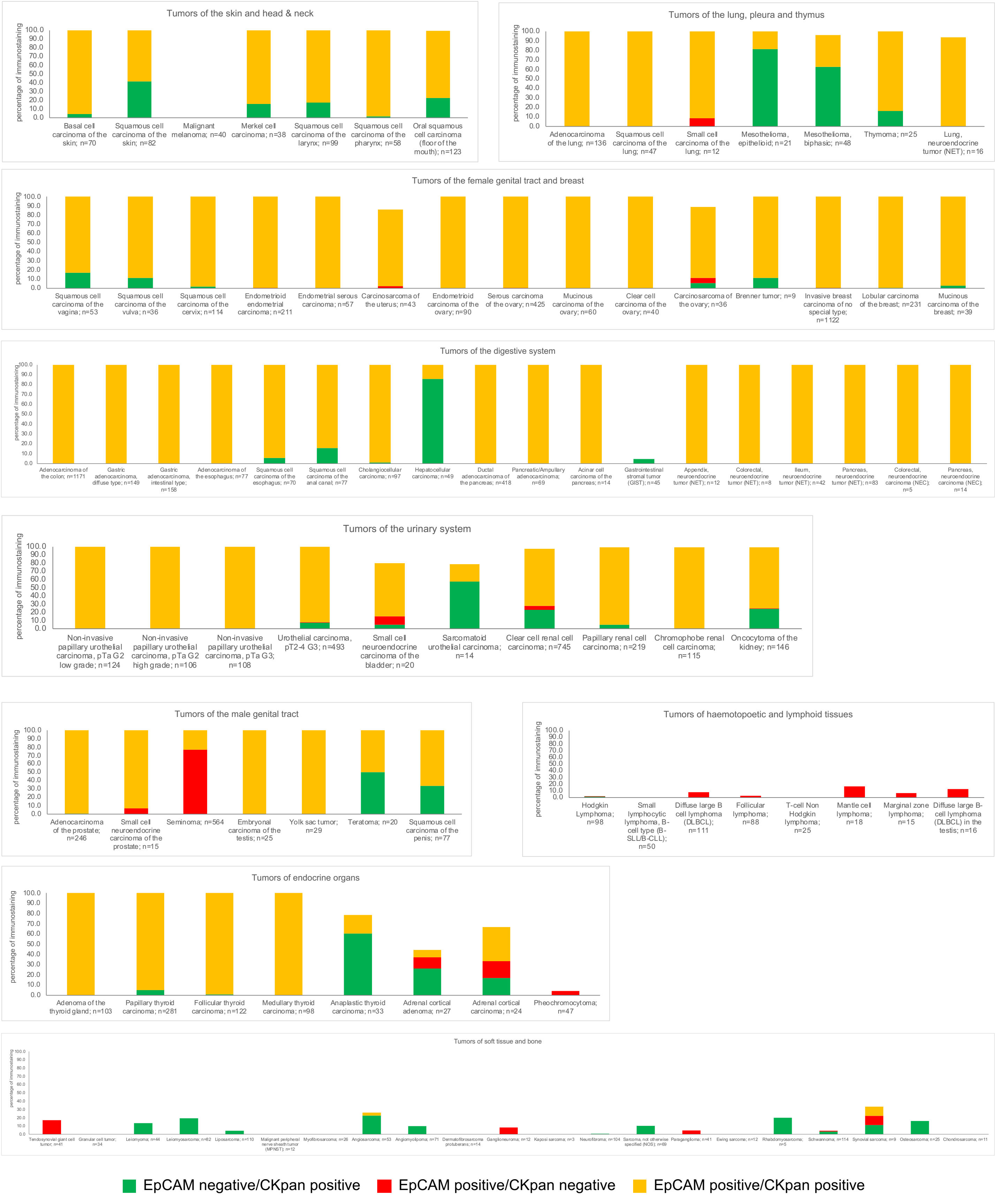

Tumors of the skin and head & neck
percentage of immunostaining
Basal cell carcinoma of the skin; n=70
Squamous cell carcinoma of the skin; n=82
Malignant melanoma; n=40
Merkel cell carcinoma; n=38
Squamous cell carcinoma of the larynx; n=99
Squamous cell carcinoma of the pharynx; n=58
Oral squamous cell carcinoma (floor of the mouth); n=123
Tumors of the lung, pleura and thymus
Adenocarcinoma of the lung; n=136
Squamous cell carcinoma of the lung; n=47
Small cell carcinoma of the lung; n=12
Mesothelioma, epithelioid; n=21
Mesothelioma, biphasic; n=48
Thymoma; n=25
Lung, neuroendocrine tumor (NET); n=16
Tumors of the female genital tract and breast
Squamous cell carcinoma of the vagina; n=53
Squamous cell carcinoma of the vulva; n=36
Squamous cell carcinoma of the cervix; n=114
Endometrioid endometrial carcinoma; n=211
Endometrial serous carcinoma; n=57
Carcinosarcoma of the uterus; n=43
Endometrioid carcinoma of the ovary; n=90
Serous carcinoma of the ovary; n=425
Mucinous carcinoma of the ovary; n=60
Clear cell carcinoma of the ovary; n=40
Carcinosarcoma of the ovary; n=36
Brenner tumor; n=9
Invasive breast carcinoma of no special type; n=1122
Lobular carcinoma of the breast; n=231
Mucinous carcinoma of the breast; n=39
Tumors of the digestive system
Adenocarcinoma of the colon; n=1171
Gastric adenocarcinoma, diffuse type; n=149
Gastric adenocarcinoma, intestinal type; n=158
Adenocarcinoma of the esophagus; n=77
Squamous cell carcinoma of the esophagus; n=70
Squamous cell carcinoma of the anal canal; n=77
Cholangiocellular carcinoma; n=97
Hepatocellular carcinoma; n=49
Ductal adenocarcinoma of the pancreas; n=418
Pancreatic/Ampullary adenocarcinoma; n=69
Acinar cell carcinoma of the pancreas; n=14
Gastrointestinal stromal tumor (GIST); n=45
Appendix, neuroendocrine tumor (NET); n=12
Colorectal, neuroendocrine tumor (NET); n=8
Ileum, neuroendocrine tumor (NET); n=42
Pancreas, neuroendocrine tumor (NET); n=83
Colorectal, neuroendocrine carcinoma (NEC); n=5
Pancreas, neuroendocrine carcinoma (NEC); n=14
Tumors of the urinary system
Non-invasive papillary urothelial carcinoma, pTa G2 low grade; n=124
Non-invasive papillary urothelial carcinoma, pTa G2 high grade; n=106
Non-invasive papillary urothelial carcinoma, pTa G3; n=108
Urothelial carcinoma, pT2-4 G3; n=493
Small cell neuroendocrine carcinoma of the bladder; n=20
Sarcomatoid urothelial carcinoma; n=14
Clear cell renal cell carcinoma; n=745
Papillary renal cell carcinoma; n=219
Chromophobe renal cell carcinoma; n=115
Oncocytoma of the kidney; n=146
Tumors of the male genital tract
Adenocarcinoma of the prostate; n=246
Small cell neuroendocrine carcinoma of the prostate; n=15
Seminoma; n=564
Embryonal carcinoma of the testis; n=25
Yolk sac tumor; n=29
Teratoma; n=20
Squamous cell carcinoma of the penis; n=77
Tumors of haemotopoetic and lymphoid tissues
Hodgkin Lymphoma; n=98
Small lymphocytic lymphoma, B-cell type (B-SLL/B-CLL); n=50
Diffuse large B cell lymphoma (DLBCL); n=111
Follicular lymphoma; n=88
T-cell Non Hodgkin lymphoma; n=25
Mantle cell lymphoma; n=18
Marginal zone lymphoma; n=15
Diffuse large B-cell lymphoma (DLBCL) in the testis; n=16
Tumors of endocrine organs
Adenoma of the thyroid gland; n=103
Papillary thyroid carcinoma; n=281
Follicular thyroid carcinoma; n=122
Medullary thyroid carcinoma; n=98
Anaplastic thyroid carcinoma; n=33
Adrenal cortical adenoma; n=27
Adrenal cortical carcinoma; n=24
Pheochromocytoma; n=47
Tumors of soft tissue and bone
Tendosynovial giant cell tumor; n=41
Granular cell tumor; n=34
Leiomyoma; n=44
Leiomyosarcoma; n=82
Liposarcoma; n=110
Malignant peripheral nerve sheath tumor (MPNST); n=12
Myofibrosarcoma; n=26
Angiosarcoma; n=53
Angiomyolipoma; n=71
Dermatofibrosarcoma protuberans; n=14
Ganglioneuroma; n=12
Kaposi sarcoma; n=3
Neurofibroma; n=104
Sarcoma, not otherwise specified (NOS); n=69
Paraganglioma; n=41
Ewing sarcoma; n=12
Rhabdomyosarcoma; n=5
Schwannoma; n=114
Synovial sarcoma; n=9
Osteosarcoma; n=25
Chondrosarcoma; n=11
EpCAM negative/CKpan positive
EpCAM positive/CKpan negative
EpCAM positive/CKpan positive
