# Supplementary material for: Epithelial Cell Adhesion Molecule (EpCAM) Expression in Human Tumors: A Comparison with Pan-Cytokeratin and TROP2 in 14,832 Tumors
Source: Diagnostics (Basel). 2024 May 17;14(10):1044. doi: 10.3390/diagnostics14101044 (PMC11120328; doi:10.3390/diagnostics14101044)
Supplement: Supplementary file 1 [file diagnostics-14-01044-s001.zip › Supplementary Figure 5 .pdf]

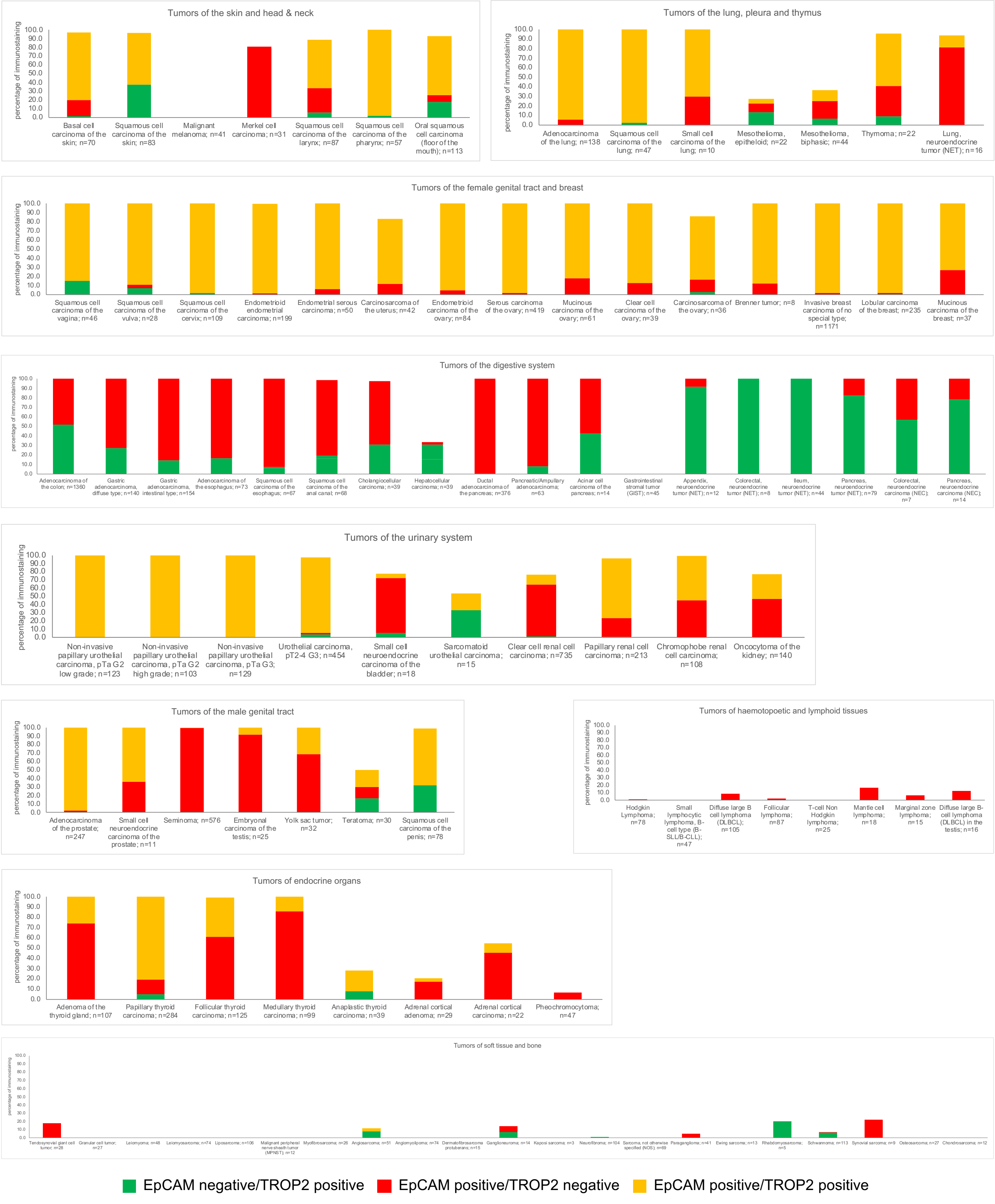
Tumors of the skin and head & neck
percentage of immunostaining
Basal cell carcinoma of the skin; n=70
Squamous cell carcinoma of the skin; n=83
Malignant melanoma; n=41
Merkel cell carcinoma; n=31
Squamous cell carcinoma of the larynx; n=87
Squamous cell carcinoma of the pharynx; n=57
Oral squamous cell carcinoma (floor of the mouth); n=113
Tumors of the lung, pleura and thymus
percentage of immunostaining
Adenocarcinoma of the lung; n=138
Squamous cell carcinoma of the lung; n=47
Small cell carcinoma of the lung; n=10
Mesothelioma, epitheloid; n=22
Mesothelioma, biphasic; n=44
Thymoma; n=22
Lung, neuroendocrine tumor (NET); n=16
Tumors of the female genital tract and breast
percentage of immunostaining
Squamous cell carcinoma of the vagina; n=46
Squamous cell carcinoma of the vulva; n=28
Squamous cell carcinoma of the cervix; n=109
Endometrioid endometrial carcinoma; n=199
Endometrial serous carcinoma; n=50
Carcinosarcoma of the uterus; n=42
Endometrioid carcinoma of the ovary; n=84
Serous carcinoma of the ovary; n=419
Mucinous carcinoma of the ovary; n=61
Clear cell carcinoma of the ovary; n=39
Carcinosarcoma of the ovary; n=36
Brenner tumor; n=8
Invasive breast carcinoma of no special type; n=1171
Lobular carcinoma of the breast; n=235
Mucinous carcinoma of the breast; n=37
Tumors of the digestive system
percentage of immunostaining
Adenocarcinoma of the colon; n=1360
Gastric adenocarcinoma, diffuse type; n=140
Gastric adenocarcinoma, intestinal type; n=154
Adenocarcinoma of the esophagus; n=73
Squamous cell carcinoma of the esophagus; n=67
Squamous cell carcinoma of the anal canal; n=68
Cholangiocellular carcinoma; n=39
Hepatocellular carcinoma; n=39
Ductal adenocarcinoma of the pancreas; n=376
Pancreatic/Ampullary adenocarcinoma; n=63
Acinar cell carcinoma of the pancreas; n=14
Gastrointestinal stromal tumor (GIST); n=45
Appendix, neuroendocrine tumor (NET); n=12
Colorectal, neuroendocrine tumor (NET); n=8
Ileum, neuroendocrine tumor (NET); n=44
Pancreas, neuroendocrine tumor (NET); n=79
Colorectal, neuroendocrine carcinoma (NEC); n=7
Pancreas, neuroendocrine carcinoma (NEC); n=14
Tumors of the urinary system
percentage of immunostaining
Non-invasive papillary urothelial carcinoma, pTa G2 low grade; n=123
Non-invasive papillary urothelial carcinoma, pTa G2 high grade; n=103
Non-invasive papillary urothelial carcinoma, pTa G3; n=129
Urothelial carcinoma, pT2-4 G3; n=454
Small cell neuroendocrine carcinoma of the bladder; n=18
Sarcomatoid urothelial carcinoma; n=15
Clear cell renal cell carcinoma; n=735
Papillary renal cell carcinoma; n=213
Chromophobe renal cell carcinoma; n=108
Oncocytoma of the kidney; n=140
Tumors of the male genital tract
percentage of immunostaining
Adenocarcinoma of the prostate; n=247
Small cell neuroendocrine carcinoma of the prostate; n=11
Seminoma; n=576
Embryonal carcinoma of the testis; n=25
Yolk sac tumor; n=32
Teratoma; n=30
Squamous cell carcinoma of the penis; n=78
Tumors of haemotopoetic and lymphoid tissues
percentage of immunostaining
Hodgkin Lymphoma; n=78
Small lymphocytic lymphoma, B-cell type (B-SLL/B-CLL); n=47
Diffuse large B cell lymphoma (DLBCL); n=105
Follicular lymphoma; n=87
T-cell Non Hodgkin lymphoma; n=25
Mantle cell lymphoma; n=18
Marginal zone lymphoma; n=15
Diffuse large B-cell lymphoma (DLBCL) in the testis; n=16
Tumors of endocrine organs
percentage of immunostaining
Adenoma of the thyroid gland; n=107
Papillary thyroid carcinoma; n=284
Follicular thyroid carcinoma; n=125
Medullary thyroid carcinoma; n=99
Anaplastic thyroid carcinoma; n=39
Adrenal cortical adenoma; n=29
Adrenal cortical carcinoma; n=22
Pheochromocytoma; n=47
Tumors of soft tissue and bone
percentage of immunostaining
Tendosynovial giant cell tumor; n=28
Granular cell tumor; n=27
Leiomyoma; n=48
Leiomyosarcoma; n=74
Liposarcoma; n=106
Malignant peripheral nerve sheath tumor (MPNST); n=12
Myofibrosarcoma; n=26
Angiosarcoma; n=51
Angiomyolipoma; n=74
Dermatofibrosarcoma protuberans; n=15
Ganglioneuroma; n=14
Kaposi sarcoma; n=3
Neurofibroma; n=104
Sarcoma, not otherwise specified (NOS); n=69
Paraganglioma; n=41
Ewing sarcoma; n=13
Rhabdomyosarcoma; n=5
Schwannoma; n=113
Synovial sarcoma; n=9
Osteosarcoma; n=27
Chondrosarcoma; n=12
EpCAM negative/TROP2 positive
EpCAM positive/TROP2 negative
EpCAM positive/TROP2 positive
