# Supplementary material for: Epithelial Cell Adhesion Molecule (EpCAM) Expression in Human Tumors: A Comparison with Pan-Cytokeratin and TROP2 in 14,832 Tumors
Source: Diagnostics (Basel). 2024 May 17;14(10):1044. doi: 10.3390/diagnostics14101044 (PMC11120328; doi:10.3390/diagnostics14101044)
Supplement: Supplementary file 1 [file diagnostics-14-01044-s001.zip › Supplementary Figure 6.pdf]

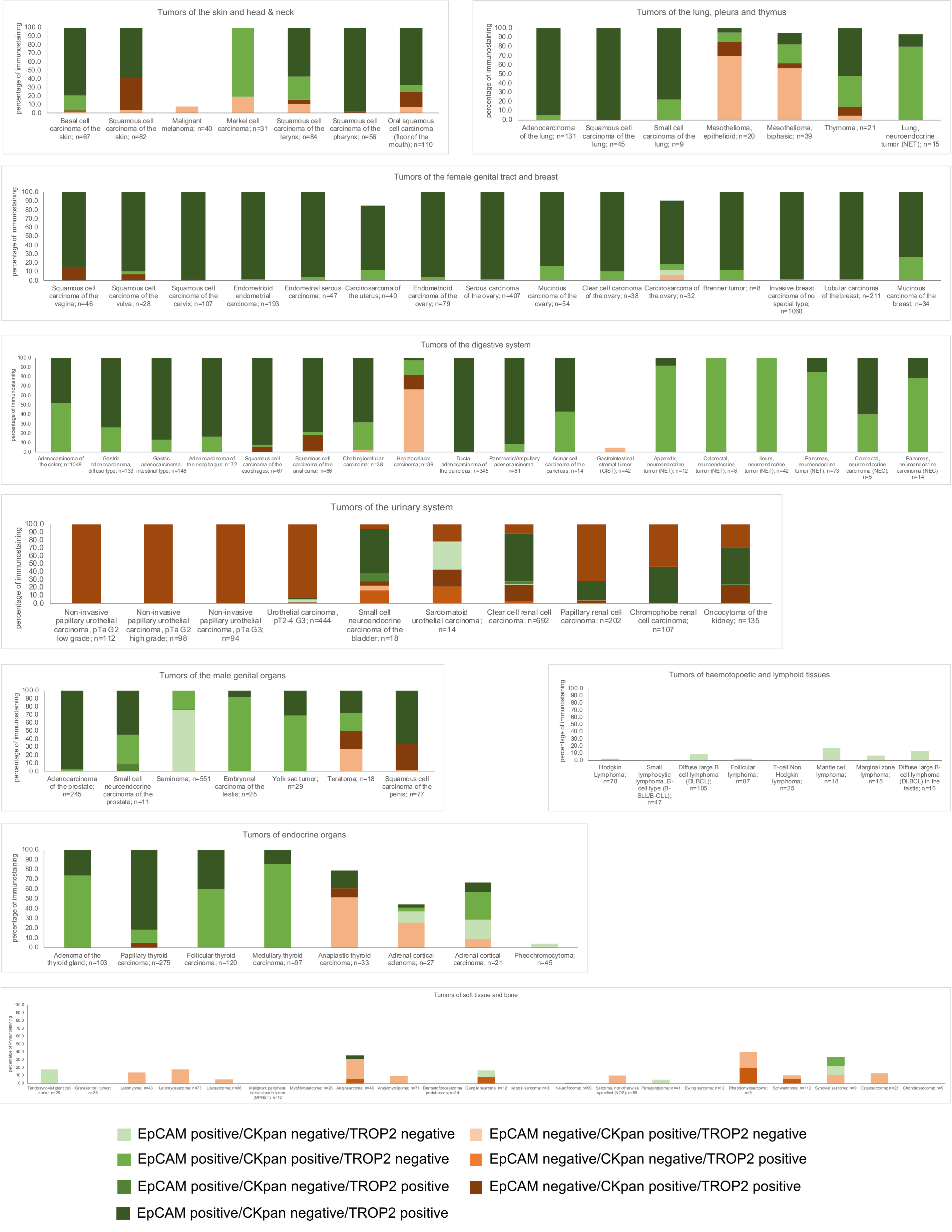
Tumors of the skin and head & neck
percentage of immunostaining
Basal cell carcinoma of the skin; n=67
Squamous cell carcinoma of the skin; n=82
Malignant melanoma; n=40
Merkel cell carcinoma; n=31
Squamous cell carcinoma of the larynx; n=84
Squamous cell carcinoma of the pharynx; n=56
Oral squamous cell carcinoma (floor of the mouth); n=110
Tumors of the lung, pleura and thymus
percentage of immunostaining
Adenocarcinoma of the lung; n=131
Squamous cell carcinoma of the lung; n=45
Small cell carcinoma of the lung; n=9
Mesothelioma, epithelioid; n=20
Mesothelioma, biphasic; n=39
Thymoma; n=21
Lung, neuroendocrine tumor (NET); n=15
Tumors of the female genital tract and breast
percentage of immunostaining
Squamous cell carcinoma of the vagina; n=46
Squamous cell carcinoma of the vulva; n=28
Squamous cell carcinoma of the cervix; n=107
Endometrioid endometrial carcinoma; n=193
Endometrial serous carcinoma; n=47
Carcinosarcoma of the uterus; n=40
Endometrioid carcinoma of the ovary; n=79
Serous carcinoma of the ovary; n=407
Mucinous carcinoma of the ovary; n=54
Clear cell carcinoma of the ovary; n=38
Carcinosarcoma of the ovary; n=32
Brenner tumor; n=8
Invasive breast carcinoma of no special type; n=1060
Lobular carcinoma of the breast; n=211
Mucinous carcinoma of the breast; n=34
Tumors of the digestive system
percentage of immunostaining
Adenocarcinoma of the colon; n=1048
Gastric adenocarcinoma, diffuse type; n=133
Gastric adenocarcinoma, intestinal type; n=148
Adenocarcinoma of the esophagus; n=72
Squamous cell carcinoma of the esophagus; n=67
Squamous cell carcinoma of the anal canal; n=66
Cholangiocellular carcinoma; n=38
Hepatocellular carcinoma; n=39
Ductal adenocarcinoma of the pancreas; n=345
Pancreatic/Ampullary adenocarcinoma; n=61
Acinar cell carcinoma of the pancreas; n=14
Gastrointestinal stromal tumor (GIST); n=42
Appendix, neuroendocrine tumor (NET); n=12
Colorectal, neuroendocrine tumor (NET); n=8
Ileum, neuroendocrine tumor (NET); n=42
Pancreas, neuroendocrine tumor (NET); n=73
Colorectal, neuroendocrine carcinoma (NEC); n=5
Pancreas, neuroendocrine carcinoma (NEC); n=14
Tumors of the urinary system
percentage of immunostaining
Non-invasive papillary urothelial carcinoma, pTa G2 low grade; n=112
Non-invasive papillary urothelial carcinoma, pTa G2 high grade; n=98
Non-invasive papillary urothelial carcinoma, pTa G3; n=94
Urothelial carcinoma, pT2-4 G3; n=444
Small cell neuroendocrine carcinoma of the bladder; n=18
Sarcomatoid urothelial carcinoma; n=14
Clear cell renal cell carcinoma; n=692
Papillary renal cell carcinoma; n=202
Chromophobe renal cell carcinoma; n=107
Oncocytoma of the kidney; n=135
Tumors of the male genital organs
percentage of immunostaining
Adenocarcinoma of the prostate; n=245
Small cell neuroendocrine carcinoma of the prostate; n=11
Seminoma; n=551
Embryonal carcinoma of the testis; n=25
Yolk sac tumor; n=29
Teratoma; n=18
Squamous cell carcinoma of the penis; n=77
Tumors of haemotopoetic and lymphoid tissues
percentage of immunostaining
Hodgkin Lymphoma; n=78
Small lymphocytic lymphoma, B-cell type (B-SLL/B-CLL); n=47
Diffuse large B cell lymphoma (DLBCL); n=105
Follicular lymphoma; n=87
T-cell Non Hodgkin lymphoma; n=25
Mantle cell lymphoma; n=18
Marginal zone lymphoma; n=15
Diffuse large B-cell lymphoma (DLBCL) in the testis; n=16
Tumors of endocrine organs
percentage of immunostaining
Adenoma of the thyroid gland; n=103
Papillary thyroid carcinoma; n=275
Follicular thyroid carcinoma; n=120
Medullary thyroid carcinoma; n=97
Anaplastic thyroid carcinoma; n=33
Adrenal cortical adenoma; n=27
Adrenal cortical carcinoma; n=21
Pheochromocytoma; n=45
Tumors of soft tissue and bone
percentafge of immunostaining
Tendosynovial giant cell tumor; n=28
Granular cell tumor; n=26
Leiomyoma; n=43
Leiomyosarcoma; n=73
Liposarcoma; n=96
Malignant peripheral nerve sheath tumor (MPNST); n=12
Myofibrosarcoma; n=26
Angiosarcoma; n=48
Angiomyolipoma; n=71
Dermatofibrosarcoma protuberans; n=14
Ganglioneuroma; n=12
Kaposi sarcoma; n=3
Neurofibroma; n=99
Sarcoma, not otherwise specified (NOS); n=69
Paraganglioma; n=41
Ewing sarcoma; n=12
Rhabdomyosarcoma; n=5
Schwannoma; n=112
Synovial sarcoma; n=9
Osteosarcoma; n=23
Chondrosarcoma; n=9
EpCAM positive/CKpan negative/TROP2 negative
EpCAM negative/CKpan positive/TROP2 negative
EpCAM positive/CKpan positive/TROP2 negative
EpCAM negative/CKpan negative/TROP2 positive
EpCAM positive/CKpan negative/TROP2 positive
EpCAM negative/CKpan positive/TROP2 positive
EpCAM positive/CKpan negative/TROP2 positive
