# Supplementary material for: Epithelial Cell Adhesion Molecule (EpCAM) Expression in Human Tumors: A Comparison with Pan-Cytokeratin and TROP2 in 14,832 Tumors
Source: Diagnostics (Basel). 2024 May 17;14(10):1044. doi: 10.3390/diagnostics14101044 (PMC11120328; doi:10.3390/diagnostics14101044)
Supplement: Supplementary file 1 [file diagnostics-14-01044-s001.zip › Supplementary Table 1.pdf]

| Organ system | Cell type | EpCAM staining |
|---|---|---|
| **Brain** | Cerebrum | Negative. |
| | Cerebellum | Negative. |
| **Endocrine Tissues** | Thyroid | Strong staining (+++) in epithelial cells. |
| | Parathyroid | Strong staining (+++) in epithelial cells. |
| | Adrenal gland | Weak (+) to moderate (++) membranous staining of adrenocortical cells |
| | Pituitary gland | Strong staining (+++) in epithelial cells of the adenohypophysis. Negative in neurohypophysis. |
| **Respiratory system** | Respiratory epithelium | Strong (+++) staining of epthelial cells, with lowest staining (+) in basal cell layers. |
| | Lung | Weak (+) to moderate (++) staining of pneumocytes. |
| **Gastrointestinal Tract** | Salivary glands | Strong staining (+++) in epithelial cells. |
| | Esophagus | Strong staining (+++) in epithelial cells. |
| | Stomach | Strong staining (+++) in epithelial cells. Weak staining (+) of parietal cells of the stomach, limited to the basolateral membranes. |
| | Duodenum | Strong staining (+++) in epithelial cells and Brunner's glands. |
| | Small intestine | Strong staining (+++) in epithelial cells. |
| | Colon | Strong staining (+++) in epithelial cells. |
| | Rectum | Strong staining (+++) in epithelial cells. |
| | Liver | Strong staining (+++) in epithelial cells of bile ducts. Hepatocytes are negative. |
| | Gallbladder | Strong staining (+++) in epithelial cells of the gallbladder and bile ducts. |
| | Pancreas | Strong staining (+++) in epithelial cells. |

| | | |
|---|---|---|
| **Genitourinary** | Kidney | Strong staining (+++) in distal tubuli, less intense (++) and focused to the basolateral membranes in the proximal tubuli. Staining (++) of the epithelial cells lining the Bowman capule. |
| | Urothelium | Strong staining (+++) in urothelium, but sometimes less intense in umbrella cells. |
| **Male genital** | Prostate | Strong staining (+++) in epithelial cells. |
| | Seminal vesicles | Strong staining (+++) in epithelial cells. |
| | Testis | Moderate (++) to strong (+++) staining in spermatogonia and spermatocytes. No staining of Sertoli cells and Leydig cells. |
| | Epididymis | Strong staining (+++) in epithelial cells. |
| **Female genital** | Breast | Strong staining (+++) in luminal cells of breast glands, weak (+) or absent staining in myoepithelial cells. |
| | Uterus, myometrium | Negative. |
| | Uterus, ectocervix | Weak (+) staining in the basal cell layer of squamous epithelium. |
| | Uterus endocervix | Strong staining (+++) in epithelial cells. |
| | Uterus, endometrium | Strong staining (+++) in epithelial cells. |
| | Fallopian Tube | Strong staining (+++) in epithelial cells. |
| | Ovary | Strong (+++) staining of oocytes. |
| | Placenta | Weak (+) to moderate (++) membranous staining in the cytotrophoblast. |
| | Amnion | Weak (+) membranous staining in amnion cells. |
| | Chorion | Moderate (++) membranous staining in chorion cells. |
| **Skin** | Epidermis | Negative. |
| | Hair follicles | Strong staining (+++) in epithelial cells of hair follicles. |
| | Eccrine glands | Strong staining (+++) in epithelial cells. |
| | Sebaceous glands | Strong (+++) staining in peripheral germinative cells. |

| | | |
|---|---|---|
| **Muscle/connective tissue** | Heart muscle | Negative. |
| | Skeletal muscle | Negative. |
| | Smooth muscle | Negative. |
| | Vessel walls | Negative. |
| | Fat | Negative. |
| | Stroma | Negative. |
| | Endothelium | Negative. |
| **Bone marrow/lymphoid** | Bone marrow | Negative. |
| | Lymph node | Negative. |
| | Spleen | Negative. |
| | Thymus | Weak (+) to moderate (++) staining in epithelial cells including corpuscles of Hassall’s. |
| | Tonsil | Strong staining (+++) in scattered squamous epithelial cells in tonsil crypts. |
