# Supplementary material for: Epithelial Cell Adhesion Molecule (EpCAM) Expression in Human Tumors: A Comparison with Pan-Cytokeratin and TROP2 in 14,832 Tumors
Source: Diagnostics (Basel). 2024 May 17;14(10):1044. doi: 10.3390/diagnostics14101044 (PMC11120328; doi:10.3390/diagnostics14101044)
Supplement: Supplementary file 1 [file diagnostics-14-01044-s001.zip › Supplementary Table 3.pdf]

	HPV status	n	EpCAM immunostaining result				P
			negative (%)	weak (%)	moderate (%)	strong (%)	
All squamous cell cancers	negative	286	20.3	28.7	16.1	35.0	<0.0001
	positive	240	8.3	28.8	26.7	36.3	
Oral squamous cell carcinoma	negative	65	23.1	26.2	13.8	36.9	0.1042
	positive	13	15.4	15.4	46.2	23.1	
Squamous cell carcinoma of the pharynx	negative	22	4.5	31.8	22.7	40.9	0.1898
	positive	34	0.0	14.7	23.5	61.8	
Squamous cell carcinoma of the larynx	negative	49	14.3	18.4	18.4	49.0	0.0615
	positive	9	0.0	44.4	0.0	55.6	
Squamous cell carcinoma of the cervix	negative	8	12.5	25.0	25.0	37.5	0.2029
	positive	66	0.0	25.8	31.8	42.4	
Squamous cell carcinoma of the vagina	negative	15	20.0	33.3	20.0	26.7	0.5364
	positive	15	6.7	26.7	40.0	26.7	
Squamous cell carcinoma of the vulva	negative	54	7.4	31.5	18.5	42.6	0.4257
	positive	26	3.8	46.2	23.1	26.9	
Squamous cell carcinoma of the penis	negative	30	46.7	30.0	3.3	20.0	0.0642
	positive	46	26.1	39.1	19.6	15.2	
Squamous cell carcinoma of the skin	negative	38	34.2	36.8	15.8	13.2	0.5821
	positive	1	0.0	100.0	0.0	0.0	
Squamous cell carcinoma of the anal canal	negative	5	0.0	40.0	20.0	40.0	0.5832
	positive	30	13.3	20.0	26.7	40.0	
