# Supplementary figures and images for: Epithelial Cell Adhesion Molecule (EpCAM) Expression in Human Tumors: A Comparison with Pan-Cytokeratin and TROP2 in 14,832 Tumors
Source: Diagnostics (Basel). 2024 May 17;14(10):1044. doi: 10.3390/diagnostics14101044 (PMC11120328; doi:10.3390/diagnostics14101044)

A)

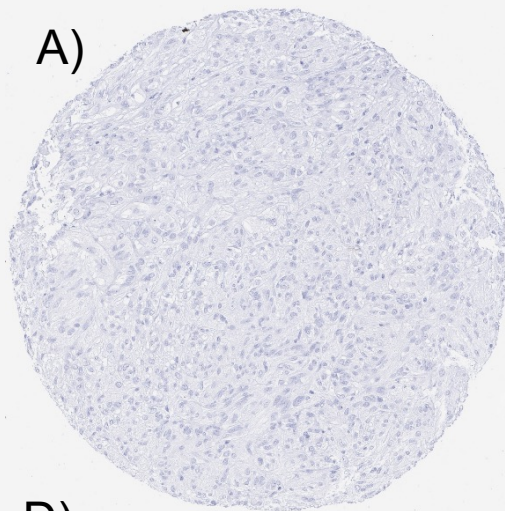

B)

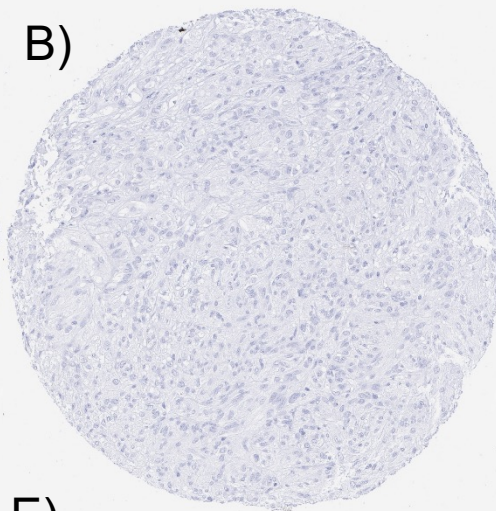

C)

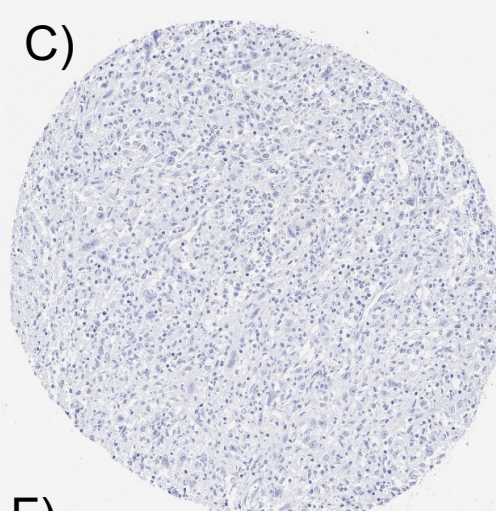

D)

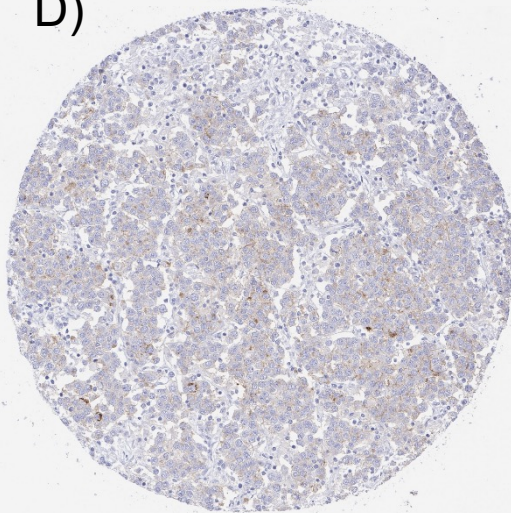

E)

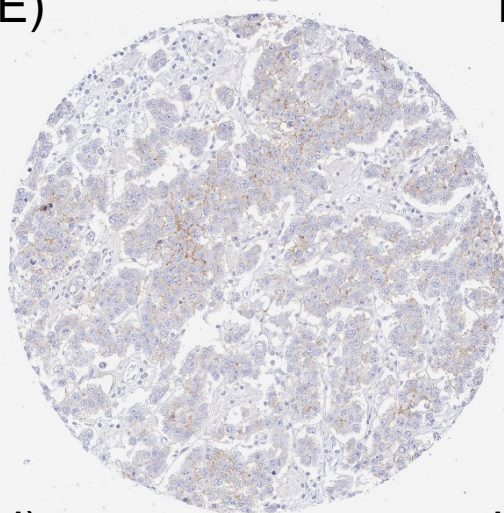

F)

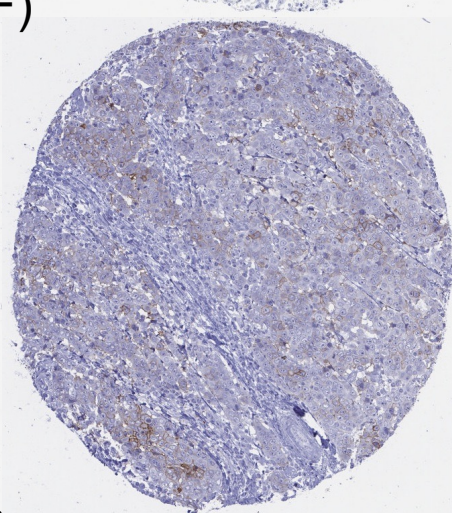

G)

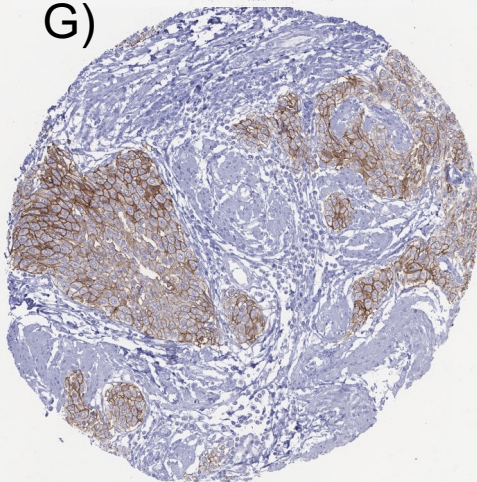

H)

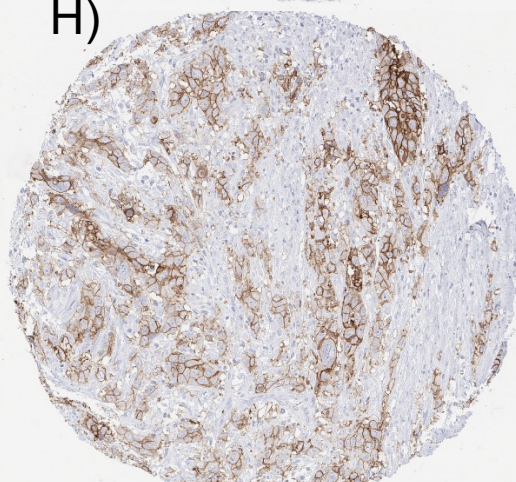

I)

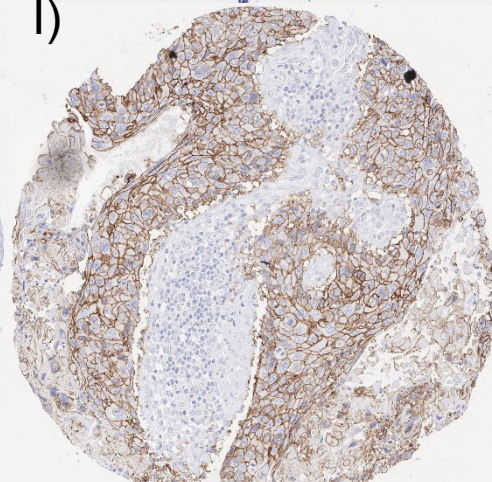

J)

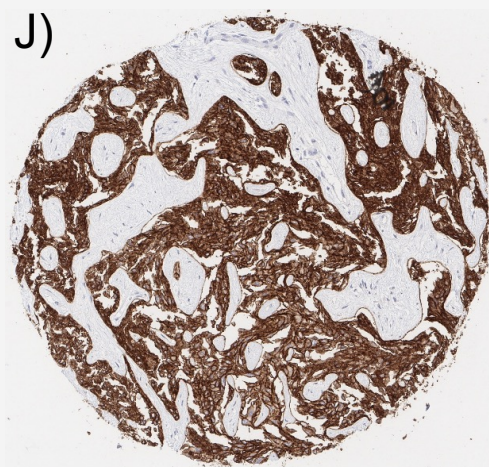

K)

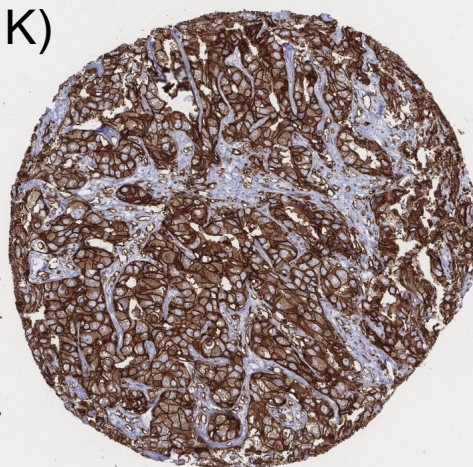

L)

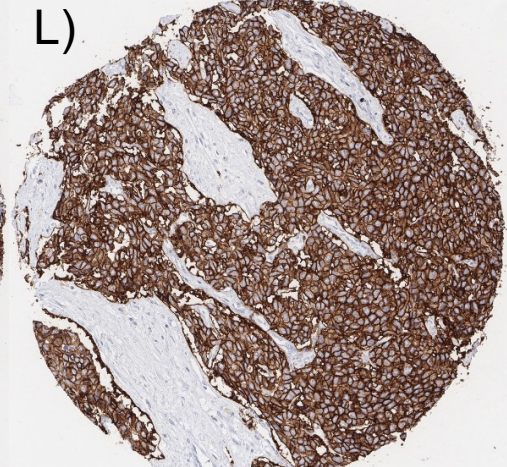

Supplement: Supplementary file 1 [file diagnostics-14-01044-s001.zip › Supplementary Figure 1.pdf]

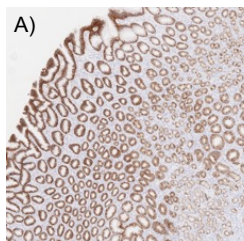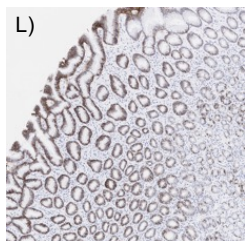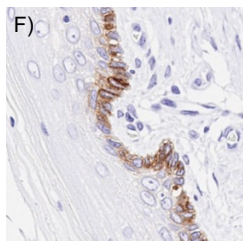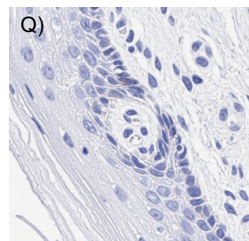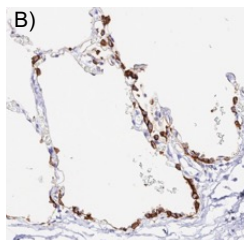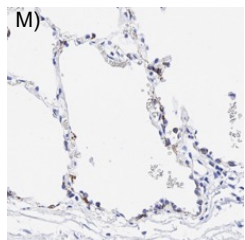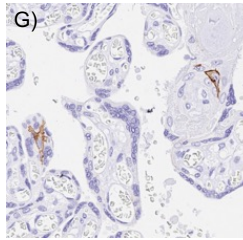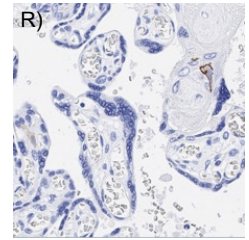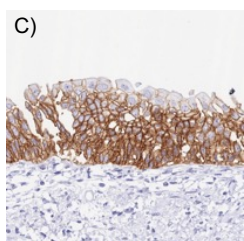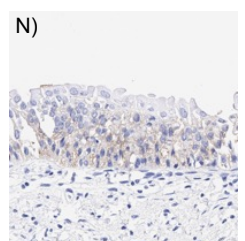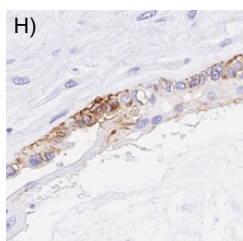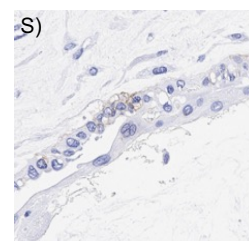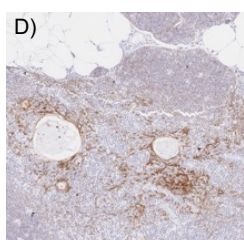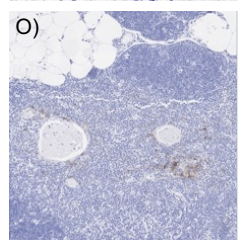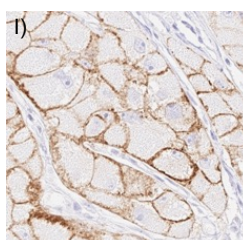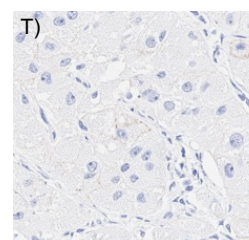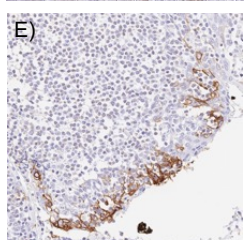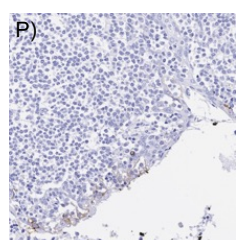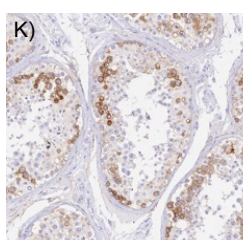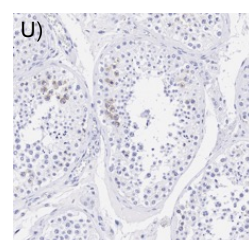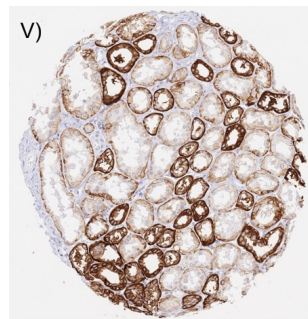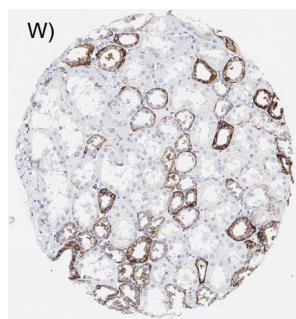

Supplement: Supplementary file 1 [file diagnostics-14-01044-s001.zip › Supplementary Figure 2.pdf]

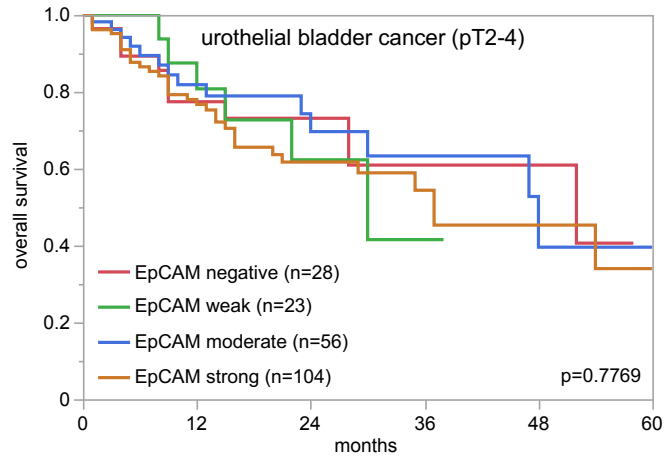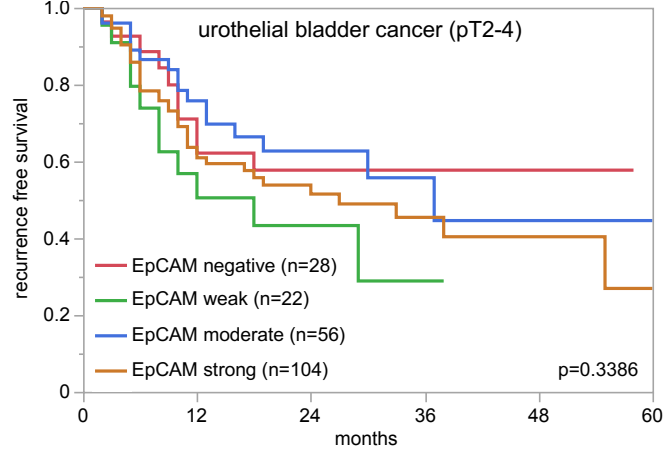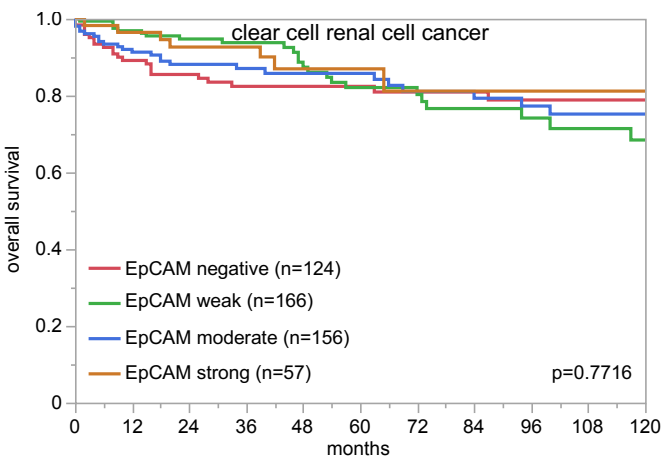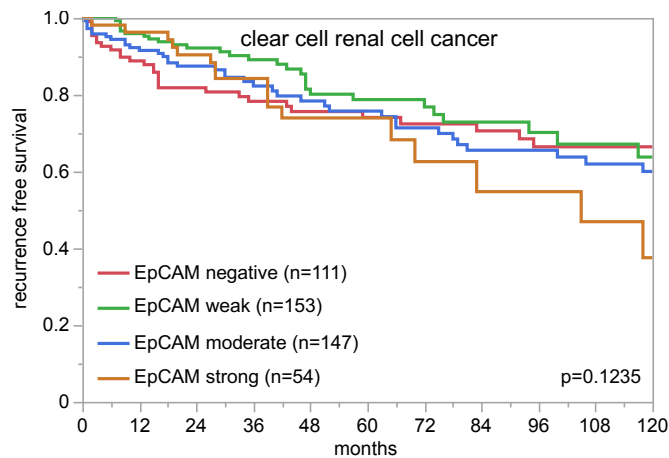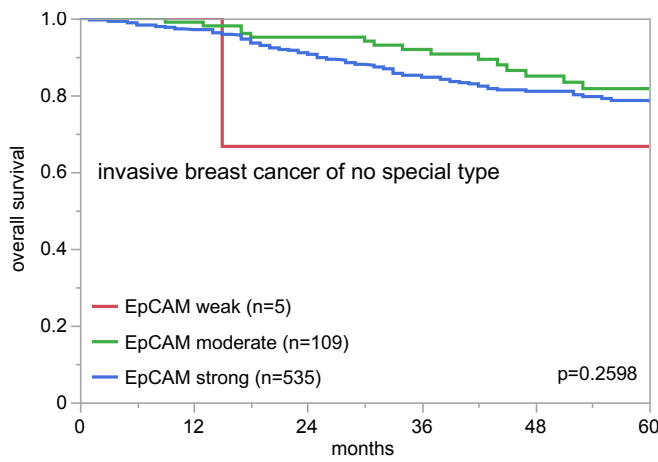

Supplement: Supplementary file 1 [file diagnostics-14-01044-s001.zip › Supplementary Figure 3.pdf]
